# Virus-Specific T Cells: Promising Adoptive T Cell Therapy Against Infectious Diseases Following Hematopoietic Stem Cell Transplantation

**DOI:** 10.34172/apb.2023.046

**Published:** 2022-11-04

**Authors:** Arsalan Jalili, Abbas Hajifathali, Mozhdeh Mohammadian, Ghazaleh Sankanian, Maryam Sayahinouri, Mahmoud Dehghani Ghorbi, Elham Roshandel, Nasser Aghdami

**Affiliations:** ^1^Department of Applied Cell Sciences, Faculty of Basic Sciences and Advanced Medical Technologies, Royan Institute, ACECR, Tehran, Iran.; ^2^Department of Stem Cells and Developmental Biology at Cell Science Research Center, Royan Institute for Stem Cell Biology and Technology, ACECR, Tehran.; ^3^Hematopoietic Stem Cell Research Center, Shahid Beheshti University of Medical Sciences, Tehran, Iran.; ^4^Department of Hematology, Faculty of Medical Sciences, Tarbiat Modares University, Tehran, Iran.; ^5^Department of Immunology, Afzalipour Faculty of Medicine, Kerman University of Medical Sciences, Kerman, Iran.; ^6^Parvaz Research Ideas Supporter institute, Tehran, Iran.; ^7^Department of Internal Medicine, Imam Hossein Hospital, School of Medicine Shahid Beheshti University of Medical Science, Tehran, Iran.; ^8^Department of Regenerative Medicine, Cell Science Research Center, Royan Institute for Stem Cell Biology and Technology, ACECR, Tehran, Iran.

**Keywords:** Post-hematopoietic stem cell transplantation infec, Adoptive T cell therapy, Virus-specific T cells

## Abstract

Hematopoietic stem cell transplantation (HSCT) is a life-saving therapy for various hematologic disorders. Due to the bone marrow suppression and its long recovery period, secondary infections, like cytomegalovirus (CMV), Epstein-Bar virus (EBV), and adenovirus (AdV), are the leading causes of morbidity and mortality in HSCT cases. Drug resistance to the antiviral pharmacotherapies makes researchers develop adoptive T cell therapies like virus-specific T cell therapy. These studies have faced major challenges such as finding the most effective T cell expansion methods, isolating the expected subtype, defining the functionality of the end-cell population, product quality control, and clinical complications after the injection. This review discusses the viral infections after HSCT, T cells characteristics during chronic viral infection, application of virus-specific T cells (VSTs) for refractory infections, standard methods for producing VSTs and their limitation, clinical experiences on VSTs, focusing on outcomes and side effects that can be helpful in decision-making for patients and further researches.

## Hematopoietic stem cell transplantation

 Allogeneic hematopoietic stem cell transplantation (allo-HSCT) is applied for treating various hematologic diseases such as aplastic anemia, paroxysmal nocturnal hemoglobinuria, acute myeloid leukemia, acute lymphoblastic leukemia, and chronic myeloid leukemia.^1^ This therapeutic approach is accompanied by dramatic damage to the immune system, causing severe immune deficiencies in HSCT recipients. The symptoms occur during the first trimester following HSCT, and the immune system recovery takes about three to six months.^2^ Due to the long recovery, secondary infections are one of the major reasons for death in HSCT cases.^3^

## Viral infections following HSCT

 About one-third of mortality-related deaths following HSCT are due to viruses like cytomegalovirus (CMV), Epstein-Bar virus (EBV), and adenovirus (AdV).^4^ Generally, CMV is not associated with specific symptoms in adults; however, it causes various complications in pregnant women and immunocompromised patients.^5^ EBV causes infectious mononucleosis associated with either non-malignant or premalignant and malignant lymphoproliferative diseases. This latent virus is usually reactivated after allo-HSCT and generates a post-transplant proliferative disease (PTLD).^6^ AdV is a highly prevalent virus that affects many children before entering school, associated with various respiratory and gastrointestinal symptoms. Similarly, this virus is quite common in patients undergoing HSCT. Although the local infections can be easily managed, the mortality rate increases in the presence of systemic infections, leading to a decrease in acquired immune response mediated by T cells and subsequent death.^7^

 Anti-EBV, anti-AdV, and anti-CMV drugs show significant effective results.^8^ However, some complications like toxicity, immune reactions, and lack of response have been observed in immunocompromised patients.^9^ Controlling resistant viral infections is mainly impressed via the capacity of the immune system restoration. Adoptive T cell therapy is a pretty effective way to restore the immune system.^10^ This immune support has been identified by assessing specific T cell responses in peripheral blood; unfortunately, there is little information about other predictive markers.

## T cell adoptive therapy

 Using T cells as biological anti-infection tools is a promising therapeutic choice because suppressing the immune system impairs T cells’ antiviral function. The leading limitations of the conventional HSCT, including the type of transplantation, human leukocyte antigen (HLA) compatibility, and patients’ background diseases, do not influence the adoptive T cell transplantation outcomes much. In this therapeutic approach, T cells must be derived from seropositive donors,^11,12^ which is easily accessible due to the abundance of CMV seropositive individuals in the community. The transplant can even be derived from third-party donors.^12^ However, T cells derived from the same donors for HSCT are more efficient than those derived from third-party donors. So far, adoptive T cell therapy has not been successful under high doses of steroids; this is one of the complications of combinational therapy that physicians should consider.^13^

 Despite the progress, lethal infections are still one of the leading reasons for morbidity and mortality following allo-HSCT. Due to the drug resistance to the current viral pharmacotherapies and the high expense of antibody therapy, researchers focus on adoptive immunotherapy with virus-specific T cells (VSTs). Clinical trials on CMV- and EBV-specific T cells confirmed their effectiveness and safety in preventing and curing these viral diseases. Moreover, multi-virus-specific T cells (MVSTs) target the most prevalent viruses following HSCT, including AdV, BK virus, and herpesvirus. VSTs can help fast reconstruction of antiviral immunity in the immunosuppressed allo-HSCT recipient. VSTs can be derived from transplant donors or third-party donors.^14^

 This study discusses T cell changes during viral infection, conventional methods for producing VSTs, different stimulatory factors for T cell expansion, using VSTs in post HSCT recurrent infections, and clinical experiences in this field.

## T cells in chronic viral infection

 Several factors can result in T cell exhaustion, like permanent antigen exposure or reduced cross-presentation to CD4 ^+^ T cells.^15^ Continuous exposure to antigens affects cell function, and antigen over-exposure or increased viral load causes more severe T cell exhaustion. Antigen exposure must last at least two weeks to one month to cause T cell exhaustion. Some other factors can also stimulate the cell exhaustion, including inhibitory receptors such as programmed cell death protein 1 (PD1), cytotoxic T-lymphocyte-associated protein 4 (CTLA4), T cell immunoglobulin and mucin domain 3 (TIM3), lymphocyte activation gene 3 protein (LAG3),^16^ and soluble molecules such as interleukin (IL)-10 (released by tumor cells) and transforming growth factor ꞵ (TGF-ꞵ).^17^ Regulatory T cells, an important source for IL-10 and TGF-ꞵ, also serve a key role in T cell exhaustion.^18^ Therefore, T cell exhaustion can be eliminated by reducing regulatory T-cells and blocking inhibitory receptors. T-cell exhaustion can also be caused by infections, especially sepsis, resulting in cytokine storms.^19^

**Figure 1:**
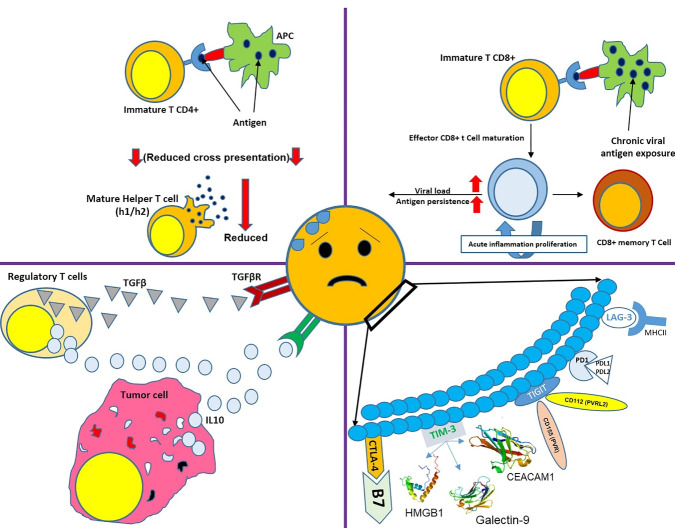
There are different factors causing cell exhaustion. This figure shows the effect of chronic viral exposure, inhibitory receptors, and their ligands, reduced cross-presentation with T CD4 + , and high levels of IL10 and TGFβ in T cells’ environment.

 Although cell exhaustion in cancer cases can be relieved by blocking the inhibitory receptors (such as PD-1 and CTLA4) and chemotherapy, this method cannot be considered effective for patients with recurrent CMV infections; since the cause of infection cannot be eliminated, and it is not possible to use blocking agents for a lifetime. Thus, it can be concluded that adoptive T cell therapy can be considered a promising approach to control recurrent viral infections in patients who underwent allo-HSCT (Figure 1).

## Common methods for producing VSTs

 Over the past 20 years, using VSTs has been significantly increased to reduce the uncontrolled proliferation of viruses in HSCT patients or other immune-suppressed patients. Numerous *in vitro* studies have been performed to reach an optimal condition and method for inducing T cell proliferation and selecting VSTs for clinical use.^20^

 In the first immunotherapy studies using VSTs, T cells were cultured with CMV lysates and CMV-infected fibroblasts, requiring cleanroom, quality control, and quality assurance to provide good manufacturing practice (GMP) grade production. Nowadays, one of the standard methods applied for VSTs production is using tetramers to select and isolate specific T cells from the patient’s entire T population.^21^ The main advantage of this method is the simplicity of the cell selection process. Besides, it does not need antigen-presenting cells (APCs), exogenous cytokines, or *ex vivo* manipulations. Additionally, since this process is performed using closed-system devices, there is no need for cleanroom and GMP equipment. However, this method only selects the T cells possessing a specific epitope for one type of HLA. Thus, it is only applicable to HLA-matched donors. Sometimes focusing on viral reactions with a particular epitope can lead to antigen escape, as observed in EBV cases.^22^

 Another method for VST isolation is based on immunomagnetic cell sorting, which is specified for isolating interferon-gamma (IFN-γ) secreting T cells created via culturing T cells with viral peptides.^23^ In addition to the fast proliferation, which is the most significant advantage, this method does not require much manipulation. This method is superior to previous ones because it can cover all viruses and antigens based on the stimulation.^20^

 Stimulating peripheral blood mononuclear cells (PBMCs) by APCs is another technique to produce GMP-grade VSTs. In 1990, this method was developed to produce EBV-specific T cells. It is initiated via stimulating CD8 ^+^ T cells with EBV^24^ and followed by coculture with dendritic cells (DCs) transduced by AdV vectors specific for CMV and EBV.^25^ Cytotoxic T cells (CTLs) generated by this method can enable T cells to detect all three viruses, including CMV (engineered from adenovirus vectors), EBV (from lymphoblastoid cell lines (LCLs)), and adenovirus (from adenovirus vectors). All the processes can be performed in a culture medium with a low blood amount (50-60 mL). This method is very time-consuming and takes long, even three months. Besides, it requires very expensive clinical viral vectors. In order to replace the viral vectors, nucleofected DCs with DNA plasmids of different viruses were used to create multi-specific VSTs. Thus, the CTLs were ready to be used after a single stimulation.^26^

**Figure 2:**
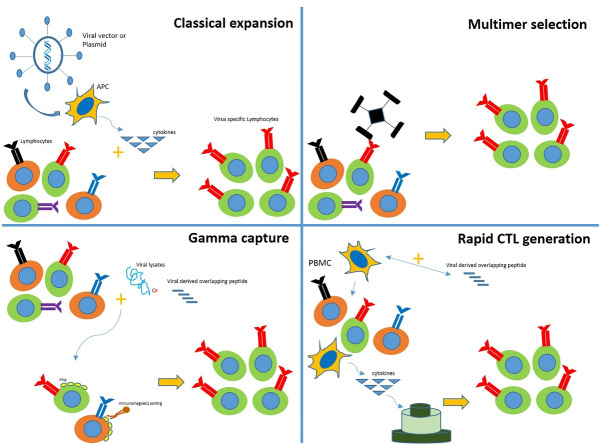
Conventional methods for producing VSTs (classical expansion, multimer selection, gamma capture, and rapid CTL generation) are illustrated in this figure.

 Despite the mentioned improvements, none of these methods can generate VSTs from seropositive donors, which is considered a major limitation. Because one of the greatest risks in viral infections is the absence of memory T cells in transplanted cells (like cells derived from the umbilical cord blood (UCB) sources or seropositive donors), leading to recipient infection infected with the pathogens.^27^ In this regard, numerous studies have tried to isolate and proliferate naive T cells from the UCB to solve this problem.^28^ Proliferating UCB-derived T cells using the G-Rex gas permeable device^29^ to sufficient amounts for clinical applications indicates the possibility of using a system that has not experienced viral conditions to produce VSTs.^30^ Clinical use of T cells to treat or prevent post-HSCT viral infections is limited by factors such as the time-consuming and complicated nature of the cell production process. Therefore, few immunotherapy centers can provide such services. Many different groups have conducted studies to overcome these limitations. Tetramer-based isolation is an up-and-coming method. However, it is costly for routine clinical applications^31^ (Figure 2).

## Different stimulation ways for T cell expansion

 The main strategy to proliferate T cells performs using magnetic beads with immobilized monoclonal antibodies to CD3^32^ or CD3 and CD28,^33^ along with cytokines. T cells in different maturation states and memory T cells show different proliferation and activation abilities; for instance, memory T cells have more proliferation potential than naive T cells. In addition, the proliferative potential of memory T cell subsets differs from central memory T cells, which show the highest proliferative rate. While T cell stimulation with the CD3 receptor (T-cell receptor) serves an important part in the differentiation fate of T cells,^34^ hemostatic cytokines are involved in proliferation, differentiation, and viability of T cells *in vivo*^35^ and *in vitro.*^36^

 IL-2 is an essential growth factor in T cell proliferation, used at concentrations of 20-1800 IU/mL.^37^ IL-7 plays a crucial part in the T cell viability and antigen-dependent proliferation of Naïve T cells.^38^ IL-15 is involved in the proliferation rate of CD8 ^+^^35^ and CD4 ^+^ memory cells in the absence of IL-7.^39^ IL-15 shares many biological features with IL-2,^40^ like stimulating the differentiation and proliferation of central memory T cells to effector memory T cells.^34^ A variety of studies that used IL-2 as a proliferation agent showed favorable results. Thus, IL-2 is considered the gold standard for stimulating T cell proliferation in most clinical trials (Table 1).

**Table 1:** Clinical trials using virus-specific T cells in post-HSCT viral infections

**Type of cell**	**Source**	**Dose**	**Expansion**	**Targeted virus**	**Transplantation**	**Infusion**	**No. of cases**	**Results and side effects**	**Ref.**
CMV- specific CD8 ^+^ clones	BM	3.3 × 10^7^–10^9^ cells/m^2^	IL-2	CMV	Allogeneic	- IV - Each week for four consecutive weeks	3	- 3/3: Prevention of viremia and pneumonia - No changes in vital signs, oximetry, chest x-ray - No GVHD	^41^
CMV- specific CD8 ^+^ clones (Phase I)	PBMC	3.3 × 10^7^–10^9^ cells/m^2^	IL-2	CMV	Allogeneic	- IV - 4 escalating doses (33, 100, and 330 million, and 1 billion cells/m^2^), - Each dose given one week apart - Over a 30-minute period - Through a Hickman catheter	11	- 11/11: CMV prevention - 4/11: No GVHD - No significant changes in vital signs, oxygen saturation, blood-chemistry, chest radiographs, and cell blood counts - 1/11: Transient fever	^42^
EBV-specific CD8 ^+^ T cells	PB	0.2–1.2 × 10^8^ cells/m^2^	IL-2	EBV	Allogeneic	- IV - 60 to 185 days post BMT	10	- 7/10: No evidence of EBV reactivation - 3/10: Evidence of uncontrolled EBV replication	^24^
EBV-specific CD8 ^+^ T cells	PB	0.2–1.0 × 10^8^ cells/m^2^	IL-2	EBV	Allogeneic/Mismatched family member or closely matched unrelated Donor	- IV - At a median of 88 days post-transplantation	39	- 39/39: 2-4 log decreases in viral DNA levels within 2-3 weeks after infusion - No lymphoma development - No toxic effects - Full response in 2 additional patients who did not received prophylaxis and developed overt immunoblastic lymphoma	^43^
CMV- specific polyclonal CD8 ^+^ and CD4 ^+^ T cells	PBMC	10^7^ cells/m^2^	IL-2	CMV	Allogeneic	- IV - In the presence of virus	8	- 5/7: Successful anti-CMV cellular therapy - 2/7: Only transient reductions in virus load - No pulmonary toxicity or other acute side effects	^44^
EBV-specific polyclonal CD8 ^+^ and CD4 ^+^ T cells (Phase I/II)	PBM	10^6^ cells/kg	IL-2	EBV	Allogeneic	- IV - Every 2 weeks, until complete remission either had (complete regression of tumor) or no response (Tumor enlargment).	5	- 3/5: Complete remission - 2/5: No clinical response - No GVHD or allo-specific antibodies - 3/5: Graft function improvement - Tumor responses in those with early, localized, polyclonal disease - 5/5: Decreased EBV load in PB to undetectable levels	^45^
CMV-specific polyclonal CD8 ^+^ and CD4 ^+^ T cells	PBMC	10^5^ cells/kg	IL-2 In-vivo expansion	CMV	Allogeneic	IV	16	- 14/16: No virus reactivation - 11/16: No GVHD - 2/16: No evaluable GVHD - 3/19: Grade I GVHD	^46^
CMV-specific CD4 ^+^ clones	PBMC	3 × 10^5^-10^6^ cells/kg	IL-2 (only from day 14 onward to ensure specificity)	CMV	Haploidentical	- IV - Between days 13 and 37 post transplantation	25	- 7/25: CMV-reactivation - No acute or chronic GvHD - No infusion-related toxicity - 5/25: Developed CMV-disease (3 eliminated infection)	^47^
CMV-specific CD8 ^+^ T cells using MHC-I-tetramers	PBMC	1.2–33 × 10^3^ cells/kg	In vivo expansion	CMV	Allogeneic	IV	9	- 8/9: Eliminated infection - 2/9: Mild GvHD (grades 1 and 2) within 2 week (both had shown grade 1 GvHD before infusion)	^21^
AdV-specific polyclonal CD8 ^+^ and CD4 ^+^ T cells	PBMC	1.2–50 × 10^3^ cells/kg	IL-2	AdV	Allogeneic	IV	9	- No acute clinical side effects - 2/9: Died due to preexisting clinical problems	^48^
CMV-, EBV-, and AdV-specific CD8 ^+^ T cells	PBMC	5 × 10^6^–1 × 10^8^ cells/m^2^	Expand in response to viral challenge after injection	AdV/ CMV/ EBV	Allogeneic (related or un-related donor)	IV	11	- 11/11: elimination of the viral pathogen - No GvHD over 3 months - No toxicities over 3 months	^25^
EBV-specific polyclonal CD8 ^+^ and CD4 ^+^ T cells (Phase II)	PBMC	2 × 10^6^ cells/kg	Weekly stimulation with autologous EBV immortalized LCLs	EBV	Allogeneic	- IV - For 4 weeks	33	- No adverse effects - 14/33: Complete remission, - 3/33 partial response - 6/33: No response at 6 months	^45^
EBV- and AdV-specific CD8 ^+^ T cells	PBMC	0.5–13.5 × 10^7^ cells/m^2^	IL-2	EBV/ CMV	Allogeneic (unrelated and haploidentical)	IV	13	- 13/13: AdV clearance - 13/13 no PTLD - No de novo GVHD	^49^
EBV-specific CD8 ^+^ T cells	PBMC	1–5 × 10^7^ cells/m^2^	Stimulation with LCLCs and IL-2	EBV	Autologous	- IV - In different doses	114	- No immediate adverse reaction - No de novo GVHD after CTL infusion - 51/114: GVHD (36: grade I; 13: grade II; 2: grade III) - 8/51: recurrent GVHD (6: grade I; 2: grade II)	^50^
CMV-specific polyclonal CD8 ^+^ andCD4 ^+^ T cells	PBMC	1.2–166 × 10^3^ cells/kg	*In vivo* expansion/ Short-term *in vitro* expansion for 2 patients (IL-2)	CMV	Transplant donor (15) Third party donor (2)	- IV - Directly after isolation, without in vitro expansion - In vitro expansion was performed for 2 cases	18	- 15/18: Responded - No acute side effects	^51^
EBV-specific CD8 ^+^ T cells using MHC-I-pentamers	PBMC	1.1 × 10^4^ cells/kg and 2 × 10^4^ cells/kg	*In vivo* expansion	EBV	Haplo-identical (mother)	IV	1	- 1/1: Complete response - No GVHD	^52^
EBV-specific polyclonal CD8 ^+^ and CD4 ^+^ T cells	PBMC	0.4–9.7 × 10^4^ cells/kg	*In vivo *expansion	EBV	Allogeneic	IV	6	- 3/6: Responded	^23^
CMV-specific polyclonal CD8 ^+^ and CD4 ^+^ T cells (Phase I/II)	Leukapheresis	3.5 × 10^4^ cells/kg	*In vivo* expansion	CMV	Allogeneic related donors	- IV - At 21 days after HSCT	18	- No infusion-related toxicity - 2/18: No need for antiviral treatment	^20^
CMV-specific CD8 ^+^ T cells using MHC-I-streptamers	PBMC	0.37-2.2 × 10^5^ cells/kg	*In vivo* expansion	CMV	Allogeneic	IV	2	- 2/2: controlled CMV virema	^53^
CMV-specific polyclonal CD8 ^+^ and CD4 ^+^ T cells (Phase I/II)	PBMC	0.9 × 10^4^-3.1 × 10^5^ cells/kg	IL-2	CMV	Autologous and Allogeneic	IV	6	- No acute adverse events - No GVHD - CMV load was disappeared	^54^
CMV-and AdV-specific CD8 ^+^ T cells using MHC-I-pentamers	PBMC	0.8–24.6 × 10^4^ cells/kg (CMV)/ 3.1 × 10^4^ and 1.7 × 10^4^ cells/kg (AdV)	*In vivo* expansion	CMV/ AdV	Allogeneic/Haploidentical (frozen donor material and third-party donors)	IV	CMV:5 AdV:8	- CMV specific T cells: 4/5 responded - AdV specific T cells: 5/6 responded - No CTL-associated GVHD	^55^
EBV-specific CD8 ^+^ T cells	PBMC	10^6^ cells/kg	Stimulation with autologous EBV transformed B cells + IL2 on day 16	EBV	Allogeneic	- IV - 1 × 10^6^ EBV-specific CTLs/kg IV weekly for 3 weeks - One-time 0.2-1 × 10^6^ unselected CD3 + T cells/kg	19	- 13/19: Complete response - No immediate adverse reactions due to cell therapy - No de novo acute or chronic GVHD or a flare of preexisting GVHD	^56^
CMV-specific polyclonal CD8 ^+^ and CD4 ^+^ T cells (Phase II)	PBMC (T cells)/ Stem cell harvest product	2 × 10^7^ cells/m^2^	*In vitro* expansion IL-2	CMV	Allogeneic	- IV - On or after day 28 post-transplantation - Treatment was delayed in case of active GVHD, organ dysfunction or active infection	50	- Median overall survival: 76 months (95% CI 56 to 96) - 13/50: relapsed over the follow-up period - Progression free survival (PFS) at one year was 89% and at 5 years was 66% - 1/50: Transplant associated microangiopathy (TAM) -1/50: Graft failure - 2/50: Lung adenocarcinoma - 1/50: Pulmonary hypertension - 6/50: Bacterial infection - 6/50: Yeast and fungal non- - 1/50: Fatal intracerebral hemorrhage	^57^
EBV-specific polyclonal CD8 ^+^ and CD4 ^+^ T cells	Whole blood or unstimulated apheresis (PBMC)	0.15-53.8 × 10^3^ cells/kg	LG2 cells + phythemagglutinine-L + IL-2	EBV	Allogeneic	IV	10	- 7/10: Responded - No acute adverse reaction - 1/7: Transient grade I-II acute skin GVHD (15 days after the first infusion/ responded well to treatment and resolved within 3-4 weeks)	^58^
AdV-specific polyclonal CD8 ^+^ and CD4 ^+^ T cells	PBMC	10^4^ cells/kg	*In vivo* expansion	AdV	Allogeneic Haploidentical	IV	5	- 3/5: Responded - No acute infusion-related toxicities - No GVHD	^59^
CMV-, EBV-, and AdV-specific CD8 ^+^ T cells (Phase I/II)	PBMC	5 × 10^6^ – 2 × 10^7^ cells/m^2^	*In vitro* with G-Rex10	CMV/ EBV/ AdV	Allogeneic. 5 Haplo, 3 MUD, 1MMUD, and 1MRD)	IV	Total: 10 CMV: 3 AdV: 1 EBV: 2 EBV ^+^ / AdV ^+^: 2 CMV ^+^ / AdV ^+^: 2	- 8/10: Complete responses - 1/10: Mild and localized skin rash and intercurrent BK infection who showed similar rash during an earlier episode of BK reactivation, prior to CTL therapy - 9/10: No infusion-related toxicity	^55^
AdV-specific polyclonal CD8 ^+^ and CD4 ^+^ T cells	PBMC	10^4^ cells/kg	IL-15	AdV	Allogeneic	IV	2	- 1/2: Complete response - 1/2: Partial response - 1/2: Developed severe GvHD	^60^
EBV-specific polyclonal CD8 ^+^ and CD4 ^+^ T cells (Phase I/II)	PBMC	5 × 10^6^ cells/kg	T: BLCL/ Stimulation with IL-2	EBV	Allogeneic	IV	10	- 6/10: No response - 1/10: Partial response - 3/10: Complete response	^61^
AdV-specific polyclonal CD8 ^+^ and CD4 ^+^ T cells	PBMC	0.3–24 × 10^3^ cells/kg	*In vivo* expansion	AdV	Allogeneic	IV	30	- 21/30: Responded - No acute side effects	^62^
CMV-specific (Phase I)	PBMC	0.66–15.41 × 10^7^ CD8 ^+^ and 0.68–9.25 × 10^5^ CD4 ^+^	IL-2 IL-15 Anti-CD3 antibody IFNγ	CMV	Allogeneic	- IV	32	- 27/32: Responded - 3/32: Died (not due to T cell therapy) - No infusion-related side effects	^63^
CMV-specific CD8 ^+^ T cells using MHC-I-streptamers (Phase I/IIa)	PBMC (third-party donor)	6.3 × 10^6^ cells 1.4 × 10^7^ cells	*In vivo* expansion	CMV	Allogeneic (Third party donor)	- IV - Within 24 hours after selection or 72 hours after apheresis.	16	- No transfusion-related reaction - 2/16: Acute and chronic GVHD	^13^
CMV-, EBV, AdV, and varicella-zoster virus-specific CD8 ^+^ and CD4 ^+^ T cells (Phase I)	PBMC	2.0 × 10^7^ cells/m^2^	IL-2	CMV/ EBV/ AdV/ multivirus	Allogeneic (Third party donor)	- IV - At a median of 75 days post HSCT	CMV:27 EBV: 1 AdV:1 Multi-virus: 1	- CMV specific T cells: 26/27 responded - EBV specific T cells: 0/1 responded - Adv specific T cells: 1/1 responded - Multi specific T cells: 1/1 responded	^64,65^
CMV-, EBV-, AdV-specific polyclonal CD8 ^+^ and CD4 ^+^ T cells/Multi: CMV- and EBV-specific or CMV- and AdV-specific CD8 ^+^ and CD4 ^+^ T cells	Leukapheresis	CMV: 7.5–16.2 × 10^4^ cells/kg EBV: 1.8–2.3 × 10^4^ cells/kg AdV: 2.7 × 10^4^ cells/kg Multi: 3.2–4.8 × 10^4^ cells/kg	*In vivo* expansion	CMV/ EBV/ AdV	7 matched unrelated, 1 sibling, and 1 haploidentical donor	- IV - Following a premedication by antihistamines and acetaminophen	CMV: 3 EBV: 2 AdV: 1 CMVa^+^ AdV ^+^: 2 CMV ^+^ EBV ^+^: 1	- Complete response in all cases (except 1 CMV) - Treatment failure in a newly acquired viral illness. - No developing or worsening	^66^
Multivirus-Specific Cytotoxic T cells (Cohort)	PBMC	5 × 10^6^ mCTLs/m^2^	Not exactly mentioned	EBV/ CMV/ AdV/ HHV6/ BK	Allogeneic	IV	4	4/4: Responded	^67^
		1 × 10^7^ mCTLs/m^2^	Not exactly mentioned	EBV/ CMV/ AdV/ HHV6/ BK	Allogeneic	IV	4	- 1/4: General disorders - 1/4: Respiratory, thoracic and mediastinal disorders	
		2 × 10^7^ mCTLs/m^2^	Not exactly mentioned	EBV/ CMV/ AdV/ HHV6/ BK	Allogeneic	IV	13	- 1/13: Gastrointestinal disorders - 1/13: General disorders - 1/13: Infections - 1/13: Renal and urinary disorders - 1/13: Reproductive system and breast disorders	
CMV-specific CTLs	PBMC	1 × 10^6^ cells/kg	Not exactly mentioned	CMV	Allogeneic	- Bolus IV - Once a week for 3 weeks	58	- 8/58: Complete response - 50/58: No complete response - 11/58: Dead - 1/58: Hepatobiliary disorders - 2/58: Catheter related infection - 1/58: Alanine aminotransferase increased - 1/58: Aspartate aminotransferase increased - 1/58: Neutrophil count decreased - 1/58: Acidosis - 1/58: Dehydration - 1/58: Renal and urinary disorders - 7/58: Hypoxia - 1/58: Thromboembolic event	^68^
Multivirus-specific Cytotoxic T cells	PBMC	2 × 10^7^ cells/m^2^	Not exactly mentioned	AdV/ CMV/ EBV/ BKV/ HHV6	Allogeneic	IV	58	- 6/58: All-cause mortality - 1/58: Blood and lymphatic system disorders - 4/58: Gastrointestinal disorders:3/58 - 2/58: General disorders - 3/58: Multi-organ failure - 1/58: Infections - 1/58: Nervous system disorders - 1/58: Vascular disorders	^69^

Abbreviations: CMV, Cytomegalovirus; BM, Bone marrow; IV, Intravenous; GVHD, Graft versus host disease; PBMC, Peripheral blood mononuclear cells; EBV, Epstein–Barr virus; PB, Peripheral blood; DNA, Deoxyribonucleic acid; AdV, Adenovirus; LCLs, Lymphoblastoid cell lines; PTLD, Post-transplant lymphoproliferative disorder; MHC-I, Major histocompatibility complex-I; CTL, Cytotoxic T lymphocytes; BLCL, B lymphoblastoid cell lines; MUD, Matched unrelated donor; MMUD, Mismatched unrelated donor; MRD, Minimal residual disease; BKV, BK virus; HHV6, Human herpesvirus 6.

## Using VSTs in post-HSCT recurrent viral infections

 HSCT can be considered one of the best treatments for malignant blood disorders. In selecting an appropriate donor, reducing the number of cytotoxic T cells should be considered, as it can reduce the risk of graft versus host disease (GVHD). GVHD prevention strategy introduces other hematopoietic stem cell sources, like haploidentical donors or UCBCs, which still result in infection, one of the most important reasons for transplant-related mortality.^70^ CMV, EBV, and AdV are the most prevalent virus pathogens in HSCT patients.^71^

 Despite the routine use of antiviral drugs, their prescription is accompanied by limitations. First, antiviral medications may suppress the immune system^72^ and cause complications in chemotherapy and radiotherapy patients. Second, although clinical trials revealed the ability of the anti-CMV and anti-EBV drugs, the efficacy of anti-AdV drugs has not been reported in trials.^73^ Antiviral drugs, especially those applied for CMV, can cause late-onset CMV infection. As soon as antiviral therapies cease, late-onset CMV may initiate, more severe than normal, with delayed immune recovery.^74^ As a result, HSC transplanted patients with viral infections may need several courses of antiviral treatments that are very costly and may lead to drug resistance. Approximately 94% of CMV species resist ganciclovir due to their mutations in the UL97 gene.^75^ In addition, Nichols et al. reported that about one-third of allo-HSCT recipients experienced an increased viral load after antiviral therapy.^76^ Many patients show drug resistance to antiviral drugs due to their continuous use, leading to severe complications like liver encephalopathy.^77^ Therefore, adoptive immunotherapy with VSTs can be applied as one of the most attractive and creative substitutes for pharmacological antivirals.

## Clinical experience with VSTs

###  CMV-specific T cells

 The first clinical trials were implemented in the early 1990s, in which CMV-specific T cells were obtained from donors, cultured, and injected into the recipients. T cell clones derived from donors were injected into fourteen HSCT patients; no recipient showed CMV infection.^42^ Parallel to efforts to produce CMV-specific T cells, many attempts were made to reduce the *ex vivo* expansion time. Peggs et al generated VSTs by targeting IFNγ-secreting cells and managed 18 patients by pre-emptive therapy. Their findings showed that this treatment significantly benefited patients on prophylaxis regimens, as six out of the seven patients did not experience reactive CMV. However, this method appears very effective in primary infections because nine of the eleven patients who received antiviral prophylaxis needed additional antiviral drugs later. In addition, many patients showed GVHD symptoms due to the active T cells.^20^

 Using T-cells isolated by tetramers was first performed by Cobbold and colleagues. They treated nine transplant patients with CMV reactivity. Following CMV-specific T cells prescription, eight patients were treated, and two developed GVHD.^21^ These findings were supported by other clinical trials and confirmed that VST is a safe therapeutic strategy and can solve many limitations of antiviral drugs.^78^ Qasim et al treated adenoviremia in HSCT pediatric patients by isolating IFN-γ-secreting T cells. However, a third-party donor was required for two out of five patients.^51^

###  AdV -specific T cells 

 Feuchtinger et al first reported using AdV-specific T cells for HSCT patients with resistant infection. AdV-specific T cells were generated based on isolating IFN-γ -secreting T cells and proliferating by IL-2 and feeder cell stimulation. According to their results, AdV disappeared in five cases, and GVHD was seen in one case.^79^

###  EBV-specific T cells 

 Due to APCs and LCLs delivering viral antigens to T cells, the conditions in which VSTs are obtained by LCLs and APCs can be critical. EBV-specific T cells were firstly established in 1996 and 1998.^48^ In a multi-institutional study in 2009, 114 HSCT patients were candidates for adoptive EBV-specific T cell therapy. A total of 101 patients received EBV-specific CTLs, and none of them (either prophylaxis or preemptive therapy) showed recurrent PTLD or de novo GVHD symptoms.^80^ MSKCC group treated 47 HSCT patients with EBV-specific CTLs derived from an HSCT donor or a third-party donor. They reported an overall response of 68% and no GVHD symptoms.^56^ Other studies with fewer patients were performed and admitted the potency and safety of the EBV-specific T cells obtained by LCLs.^81,82^

###  Multi-virus-specific T cells

 MVSTs target the most common post-HSCT viruses. In order to enhance the specificity of CTLs against viruses and target more infections, different studies were conducted to target specific viruses, like targeting EBV and AdV,^80^ CMV and AdV,^37^ and CMV, AdV, and EBV.^25^

 HSCT recipients can also be infected with other viruses, including BK, human herpesvirus 6 (HHV6), influenza, parainfluenza, coronavirus, and respiratory syncytial virus, causing morbidity and mortality. In order to expand VSTs for other viruses, a group of scientists developed a way to create polyclonal T cells (CD4 ^+^, CD8 ^+^ ) for different viruses, including *Elizabethkingia*, CMV, Adv, BK, HHV6, respiratory syncytial virus, and influenza, to face against other post-HSCT viral risk factors.^83^

 MVSTs were also used in the clinic. The most critical inclusion criterium in all clinical trials is donor seropositivity. Clinical experiments in T-cell immunotherapy with seropositive donors are minimal, but recently UCB-derived CTLs have been assessed in phase I clinical trials. Among the nine patients who used MVSTs against CMV, EBV, and Adv, only three represented active viral reactivation. One patient showed both active CMV and AdV infection, represented increased CMV and AdV-specific T cells and decreased CMV and AdV viruses following VSTs injections, and was successfully treated without antiviral pharmacotherapy. Two patients who experienced EBV reactivation or infection before or immediately after VSTs injection were treated without using antiviral drugs, and VSTs were detectable in their peripheral blood.^84^

###  Third party donor’s T cells 

 Although the frequency of refractory infections is relatively low in HSCT recipients, which makes it unreasonable to prepare VSTs for all at-risk patients, the aggressive character of the viral pathogens demands immediate availability to VSTs in antiviral therapy non-responder patients. Waiting 8- to 10-week to generate VSTs is too long for patients who developed an infection. This obstacle can be dominated by off-the-shelf third-party T cells. Production and storage of HLA-matched VSTs from third-party donors (bio-banking) could be a promising approach for adoptive VSTs therapy for patients with no HLA-matched donor.

 Previous studies in this regard are summarised in Table 1. First, Haque et al. applied EBV-specific T cells to 33 HSCT or solid tumors surgery patients to treat their PTLD. They reported a total response of 64% and 52% in five weeks and six months, respectively. No evidence of GVHD or rejection was observed. Best results were obtained when patients received HLA-matched transplants.^45^ In 2011, Qasim et al. conducted a clinical trial on AdV-infected HSCT patients and applied VSTs obtained from third-party donors using isolating IFN-γ secretory cell method. They reported an increased risk of alloreactive T cells and GVHD.^85^ The Memorial Sloan-Kettering group evaluated the efficacy and safety of this method by comparing EBV-specific T cells and donor lymphocyte infusion (DLIs) in PTLD patients. Although the responsiveness was the same in both groups, GVHD incidence was higher in the DLI group recipients.^86^

 Finally, a multi-institutional study used previously-stored VSTs from a third-party donor and showed that the procedure was safe. According to their study, HLA incompatibility can lead to complications; in other words, the shared allele must identify the specific epitope of the virus. A professional team is required to isolate, store, and inject the virus-specific T cell products.^87^

## Conclusion

 Trials using VSTs (mono-specific or multi-virus) demonstrate their ability to cure recurrent and pharmacotherapy refractory viral infections. No side effects such as GVHD were reported, even with alternative donors. Third-party donors offer a great opportunity to use the off-the-shelf product for many post-HSCT infections. It can be a promising therapeutic approach for many post-HSCT infections. Despite the limitations in generating VSTs, they have shown promising outcomes in clinical trials.

## Competing Interests

 None.

## Ethical Approval

 Not applicable.
